# New quality regulations versus established nursing home practice: a qualitative study

**DOI:** 10.1186/1472-6955-11-7

**Published:** 2012-06-07

**Authors:** Anne Marie Sandvoll, Kjell Kristoffersen, Solveig Hauge

**Affiliations:** 1 Faculty of Health Studies, Sogn og Fjordane University College, Førde, Norway; 2 Faculty of Health and Sport Sciences, University of Agder, Kristiansand, Norway; 3 Institute of Health and Society, Department of Nursing and Health Sciences, University of Oslo, Oslo, Norway; 4 Faculty of Health and Social Studies, Telemark University College, Porsgrunn, Norway

**Keywords:** Qualitative methods, Nursing homes, Nursing practice, Regulations, And routines

## Abstract

**Background:**

Western governments have initiated reforms to improve the quality of care for nursing home residents. Most of these reforms encompass the use of regulations and national quality indicators. In the Norwegian context, these regulations comprise two pages of text that are easy to read and understand. They focus particularly on residents’ rights to plan their day-to-day life in nursing homes. However, the research literature indicates that the implementation of the new regulations, particularly if they aim to change nursing practice, is extremely challenging. The aim of this study was to further explore and describe nursing practice to gain a deeper understanding of why it is so hard to implement the new regulations.

**Methods:**

For this qualitative study, an ethnographic design was chosen to explore and describe nursing practice. Fieldwork was conducted in two nursing homes. In total, 45 nurses and nursing aides were included in participant observation, and 10 were interviewed at the end of the field study.

**Results:**

Findings indicate that the staff knew little about the new quality regulations, and that the quality of their work was guided by other factors rooted in their nursing practice. Further analyses revealed that the staff appeared to be committed to daily routines and also that they always seemed to know what to do. Having routines and always knowing what to do mutually strengthen and enhance each other, and together they form a powerful force that makes daily nursing care a taken-for-granted activity.

**Conclusion:**

New regulations are challenging to implement because nursing practices are so strongly embedded. Improving practice requires systematic and deeply rooted practical change in everyday action and thinking.

## Background

Many Western governments have initiated reforms to improve the quality of care for older people and to promote the rights of nursing home residents [1,2]. In several countries, these reforms have been developed as national movements or as national health reforms [1,3-7]. Although different countries have implemented quality improvement reforms in different ways, most share the common idea of using regulations and national quality indicators [5,7].

Similar quality improvements have occurred in Norway, where new regulations were issued for improving quality in nursing homes in 1997 [8]. These regulations required that all residents in nursing homes receive individual and fundamental care, and have meaningful activities and the right to participate in decisions concerning their own daily life situation. These regulations were revised in 2003, when user involvement was strengthened. Residents’ right to provide advice was extended to the right to participate in planning daily care [9]. This indicates that user involvement in particular is viewed as important for improving quality of care in nursing homes.

Inspections undertaken by the authorities in several nursing homes in Norway suggest that many staff in nursing homes are unaware of the new regulations. In addition, research shows that even when the staff do know about policies regarding older people’s need for influence, these are not always implemented in everyday practice. Persson and Wästerfors highlight the gap between policy and practice regarding older people’s influence in nursing homes [10]. In their study, they describe that, while staff members are aware of residents’ rights to user involvement and they see resident influence as desirable, they simultaneously formulate accounts to justify their inability to meet this ideal.

Scalzi et al. discuss the problem of bringing about changes in practice by introducing new regulations [6]. Several different models have been implemented in nursing homes [5,11-15], but Scalzi et al. claim that there is little evidence in the literature to show that new regulations improve the quality of care for residents [6]. Scott-Cawiezell et al. [16] and Wiener [7] point out that it is a big challenge to change practice by implementing new regulations. There is also a lack of information about how quality of care improvements are sustained [17].

This review of the literature indicates that it is not enough to simply establish new regulations. The question is whether other factors are of equal importance in improving quality in nursing practice. Rahman and Schnelle argue that staff interactions with residents are fundamental if improved quality of care is to be realized [1]. They suggest that direct observation of staff–resident interactions is necessary to develop a quality-of-life information base that can be used for improving these interactions.

Based on the research outlined above, we decided to further investigate why it seems so hard to implement new regulations in nursing homes. We take as our point of departure the implementation of new regulations in a Norwegian context. The Norwegian regulations consist of two pages containing specific information about how staff should meet the daily needs of nursing home residents to ensure good quality care.

The aim of this study was therefore to explore and describe nursing practice in nursing homes in order to get a deeper understanding of why it is so hard to implement new regulations.

## Methods

For this qualitative study, an ethnographic design was selected to gain an in-depth understanding of nursing home practice [18].

### Context

The context of this study was two long-term units in two nursing homes. The first nursing home (NH No. 1) lies in a small town and is a complex building consisting of three units, a cafeteria and a day care centre. There were 74 residents at the time of the study. Each resident had a private single room with bathroom and most lived there permanently. The study was conducted in the long-term unit, which had 30 residents and 30 nurses/nursing aides at the time the data were collected.

The second home (NH No. 2) is smaller and located in a rural village in a small municipality. This nursing home has two wards with a total of 46 residents, who also had private single rooms with bathrooms. The ward chosen for the study had 20 residents and 15 nurses/nursing aides.

In both nursing homes, on a typical morning shift, groups of 7-8 residents, usually in adjacent rooms, were cared for by two nursing aides with a supervising nurse administering medicines and helping where needed. Fewer staff worked on the evening and night shifts.

The residents in both nursing homes were all frail and had different physical illnesses. In addition, some of them suffered from cognitive impairment. Because of the residents’ poor health, they needed help with their morning care, during meals and with activities. Most of them needed wheelchairs for mobility.

### Participants

A total of 45 nursing aides/nurses agreed to participate in this study: 24 nursing aides and 6 nurses from NH No. 1, and 13 nursing aides and 2 nurses from NH No. 2. The participants were all female and aged between 25 and 60 years old. Two of the nurses had started working at the nursing homes within the previous two years, while the others had been working there for 10 to 20 years. In Norway, nursing aides have three years’ training in upper secondary school and require government approval to practise. Nurses undertake a three-year course at a university or university college after upper secondary school. They also require government approval to practise.

### Data collection

The first author collected data by participant observation and interviews as part of her PhD research. The data were collected from August to November 2008 in NH No. 1 and from January to March 2009 in NH No. 2.

The researcher spent about four hours in the field each day observing and communicating with the staff. Observations were carried out in residents’ private rooms, the kitchen, the living-room and staff report room. Field notes were taken during the observations and transcribed immediately.

Interviews were conducted at the end of each observation period with three nursing aides and two nurses in each unit (10 in total) who had become key informants during the observations. The interviews in NH No. 1 were initially based on a pre-developed interview guide. Examples of themes that were addressed are: What rules they thought governed the practice in the nursing home; whether they were familiar with the new regulations; how they knew how to help different residents and how they knew what to do on different shifts. In NH No. 2, initial themes from the preliminary analysis of data from NH No. 1 were further investigated and presented back to the participants to explore and validate their importance. Examples of themes that were further investigated are the importance of getting the morning care done in time, and the fact that the work appeared to flow automatically during the day.

The material for analysis covered 111 pages of (single-spaced) field notes and 107 pages of transcribed interviews.

### Analysis

In ethnographic studies, analysis is a continuous process rather than a separate step [18,19]. It starts with the literature review and does not finish until the project is completed. Analysis is iterative and circular between theory, hypothesis and data [19].

The first step in the analysis was to read the data carefully to become thoroughly familiar with them. Detailed and repeated reading was necessary to look for useful analytical themes. These themes were developed spontaneously, based on statements from the participants in the interview or the field notes: for example, the participants made statements such as “we just do it” or “the routines rule the day”. Other themes were developed by the researcher based on the participants’ statements: for example, “we just know” [18]. In step two, a detailed examination of the identified themes was undertaken to clarify and deepen our understanding of each theme. The third step involved an interpretation of the relationship between the themes [18]. For example, how could we explain that staff always knew what to do, even though they had not read any instructions? In this analytical step, different theoretical perspectives, common sense expectations and stereotypes played important roles [18]. By striving to be reflexive along with reading different theories, we finally managed to make sense of our data and gained a new understanding of the nursing practice in the nursing homes.

### Measures taken to ensure trustworthiness

Throughout the whole research process, we aimed to maintain a reflective research position as a team. Therefore, in both the field study and the interviews, the first author examined and discussed her work and developed her skills as an ethnographic researcher in dialogue with the co-authors. For example, after gaining experience with interviewing in NH No. 1, she used the interview guide in a more flexible, reflective and confident way in NH No. 2 [18]. This kind of experience in interviewing is important so that the researcher becomes capable of saying or doing what is needed at the exact moment, for example, by asking questions spontaneously without the use of an explicit plan.

Further, during the whole analytical process, the three authors discussed, questioned and argued for our interpretations to the best of our ability. Our long experience of working or visiting nursing homes made it challenging to question the obvious; we therefore asked ourselves questions such as: What does this mean? How can we understand this pattern? What if our interpretation is completely wrong?

### Ethics

The Region’s Medical Ethics Committee (REK West, University of Bergen) and the Norwegian Social Science Data Services approved the study, and it was endorsed by both nursing homes. Voluntary, informal written informed consent was obtained from all participants. The research process emphasized the principles of anonymity, protection from harm and proper data storage.

## Results

In line with the literature presented in the introduction, our study reveals that most of the staff did not know about the new regulations, with only two of the nurses knowing about them. Therefore, the regulations had little direct influence on the staff’s work. In the analysis, it appeared that more important factors were guiding the staff’s actions. Analysis revealed two main themes: commitment to daily routines and “we just know”. An overall interpretation of our findings can be summarized as “we just do it”.

### Commitment to daily routines

The informants were quite clear that routines were the most important guide for their daily work.

"We try to have routines, to get finished with the morning care and all that, that’s what rules the work (Participant 3)."

The routines function as a timetable, where specific tasks should be done at particular times. Routines such as helping each resident with morning care and meals seem to be the most important tasks for the staff during the day shift.

Every morning after a short report from the night shift (given from 7:30 a.m. to about 7:45 a.m.), the staff start to help the residents with their morning care. Each staff member usually has to assist three or four residents. Morning care implies more than just helping the residents to get up and get dressed for the day; it includes tidying the residents’ rooms, taking dirty clothes to the laundry, removing used glasses and cups, airing rooms, making beds, removing rubbish and tidying and cleaning the utility room.

After finishing this significant amount of work called “morning care”, staff seem to move on to the next task automatically, serving breakfast (from 9 a.m. to 9:30 a.m.). Breakfast is served in the dining room to those who eat together, or in residents’ own rooms to those who prefer to eat alone and to those who need help while eating. At this stage in the daily work schedule, the staff’s sole focus is on serving the meal.

"The meals rule the day, I think. I mean, we plan the work around the meals and some breaks maybe. It’s like we finish this before breakfast and that before dinner (Participant 1)."

Later on, at around 11:30 a.m., the staff have a 30-minute break to eat lunch and then they move on to a new task: taking care of the residents’ toileting needs before their dinner. To maintain the schedule for the next meal, this task also has to be timed like the morning care. In reality, routines such as morning care and meals become the rules. The commitment to covering the residents’ basic needs is a strongly developed pattern. For example, there appears to be an unwritten rule that the staff do not have their own lunch break before the residents have received their morning care and breakfast. As a minimum, all the residents have to get their morning care before dinner, which is served at 1 p.m. If staff fall behind schedule, the routines take priority and the staff’s own break time is often reduced. This pattern of performing the day’s work was similar throughout the whole observation period in both nursing homes in this study.

### “We just know”

In addition to always knowing where to start and finish the day’s work, the staff also know exactly what to do on a detailed level, which means knowing all the details of every specific routine, and residents’ different needs.

During morning care, staff move from room to room and help each resident with whatever he or she needs. This varies considerably because some residents need help with everything, while others only need guidance during morning care. The staff do not refer to written notes about what to do; they just do it automatically. For residents who are able to say what they want, many minor requests are met: how their pillows should be placed on the bed, how their hair is supposed to be styled, special private items they like to carry with them all day and so on. For example, while assisting a man to bed after dinner.

"While helping him over to the bed, they lower his trousers. The nursing aide places a towel on the bed stand and then she gets two urine bottles and places them on top of the towel. Afterwards, in the corridor I say to her that it seems like she remembers all the details very well, for example to lower his trousers before he gets into bed to have a nap after dinner. “I just know,” she says. “It’s not written anywhere?” I ask her. “No, he likes it that way, and it’s also important that we empty his bottles often, he doesn’t like them getting too full,” she said (Field notes 9)."

One nursing aide told me that doing these little things did not cost her anything, and they were not done to get a reward or anything; she just does them. It appears to us that the staff’s specific knowledge about each resident’s individual wishes was closely connected with their long working experience

"It’s because we know them very well, because we have been working here for a long time, and every resident has things they like or not, and many of them like to have routines, some of them don’t like to get up before breakfast, others like that, so we learn to know how they like it (Participant 3)."

In situations with new residents or residents who are not able to state their wishes, staff usually ask the residents or their relatives about their needs and habits, and then pass on this information to the rest of the staff. The staff seldom write down or read procedures for residents, but this does not mean that they do not communicate about the care they provide.

"We get a lot of information about the residents during the oral report. And, for example, like him…, that’s really something we just know, because he was going home, and we had talked about it because he had been in bed for three months, so we talked a lot about when we could start to get him up walking again. We discuss and talk a lot about it…, so that’s why everybody knew that they had to practise walking with him (Participant 9)."

For residents’ meals, there was written information about each resident’s food preferences, but the staff tended to know the preferences without reading the notes. In addition, they were very creative in using different techniques to help residents who struggled to eat for various reasons.

"It becomes like that after knowing them for a while, but we have to get to know them, that takes some time. And we also learn from each other, from how others do it, this looks nice, maybe I can do it like that too. We watch the little details others do. I feel like I learn a lot from the nursing aides who have worked here a long time (Participant 4)."

This shows that staff in the nursing homes seem to know all the residents very well. It also shows how closely staff work with each other, and that the usual patterns of work seem to be incorporated in each and every one of them over time.

### “We just do it”: an overall interpretation

The two main findings in this study indicate that “commitment to daily routines” and “we just know” are of great importance for care practice. During analyses, these two themes became very obvious as strong patterns that guided nursing home practice. Routine and always knowing what to do mutually strengthen and enhance each other, and together they are a powerful force that makes daily nursing care taken for granted. We have presented them as two separate themes in this paper, but it is important to underline that in practice they are closely interwoven and together govern the staff’s practice. When staff are asked to describe this practice, they are not able to do so. Therefore, our overall interpretation is “we just do it”.

## Discussion

The findings in this study indicate that the new regulations have limited importance, and that daily work in nursing homes is ruled by other factors. First, in our study it became very clear that the staff in the two nursing homes were not familiar with the new regulations, possibly because they had never been informed about them. The problem of information flow from policymakers to those who put the policies into practice is well known [20,21]. In the Norwegian case, action was taken to overcome this problem by making the regulations short (only two pages) and easy to read and understand, and by distributing them to all local authorities. Despite these efforts, the staff did not know about the regulations. However, even if the staff had read and understood the regulations, studies indicate that knowledge about regulations is not sufficient to effect changes in nursing practice [7,16,22]. Despite staff lacking knowledge about the new regulations, we actually saw many examples of good practice that satisfied the requirements of the regulations. Our findings reveal, for instance, that staff fulfilled the wishes of many residents in relation to the details of their care. Similar findings are described in studies by Dunér and Nordström and Sacco-Peterson and Borell [23,24].

If the staff do not know the regulations, how is it that in many cases residents are given the right to decide matters of daily care? This might be because the regulations mirror values that are already of importance in our cultural collective. It is therefore reasonable to claim that values such as user participation influence the staff in nursing homes. In addition, similar values may have been embedded in nursing for years, demonstrated in concepts such as getting to know patients in order to deliver person-centred practice and optimize participation [25-27]. This might explain why the staff were able to meet the requirements of the new regulations but were unaware of doing so.

Second, our study shows that routines are important structuring elements for the work in nursing homes; they help staff to get the care done and make the day predictable for themselves and for residents. The strength and importance of daily routines have been described in a range of empirical studies, for instance Boge [28], Christiansen [29], Harnett [30], Persson and Wästerfors [10], Sellerberg [31], and Zisberg et al. [32]. Routines in nursing homes might be considered rigid and as having negative consequences for residents [32], especially if they dominate practice and reduce residents’ influence [26]. The opportunity for participation or influence depends on staff routines, whether it is a “good match” or if it “disrupts” or “disturbs” the staff in their routines [33]. On the other hand, routines might also be beneficial. Research confirms that daily routines facilitate well-being in older people [32], and that residents are pleased to live according to “house rules” [34]. For instance, residents often want to get up and be dressed before breakfast, and if something upsets the schedule, residents may remark on it [31]. This indicates that routines can have benefits for nursing home practice; nevertheless, they should never function as a predictor of qualitatively good care.

Third, in addition to routines, our study highlights that the staff always knew what to do. Schirm et al. described a similar pattern [35]; the findings in their study showed how nursing staff “just got to know how”, and that staff over time developed skills that were just taken for granted. But how do staff actually know what to do in their daily work? How do they know all the details and what to do all the time? To explore this issue and understand it better, Bourdieu’s concept of habitus seems helpful. Bourdieu suggests that it is not sense, or theories, or systems of regulations that govern practice, but the practical sense itself, which is creative, unpredictable and improvising [36,37]. Habitus is a collective pattern of thinking and acting, and is often taken for granted within a group [38]. Members of a group act according to a practical sense, and when asked to describe the rules they act under, they are unable to do so [36,39]. Habitus regulates actions without being a product of rules; it makes a group of people act as though “collectively orchestrated without the action of a conductor” [36]. We think that in the nursing homes we studied, the staff have developed a habitus of caring. This might have something to do with their education and training, and with the fact that they have worked in this way for a long time, as several of the staff have been employed there for 15 to 20 years.

Fourth, our overall understanding in this study is “we just do it”. We interpret this as a combination of the staff’s habitus and the tradition of daily routines. It appears that habitus and routines form a powerful “team” and, to our knowledge, this combination has not previously been described.

### What can we learn from our findings?

We know that it is a challenge to change the culture or practice in any organization. Our point is not that regulations are a waste of time and money because the staff do not know about them, but rather that, despite their importance, it is naive to expect regulations alone to be an effective instrument in driving change.

When implementing new regulations, authorities and work-place managers must clearly communicate the new vision that changes are designed to create [40] and ensure staff participate in the change process [40,41]. Furthermore, Berkhout et al. found that conditions for successful implementation were important at the ward level in particular, and that more attention should be given to educating nursing staff about changes [22]. The findings in our study support the need for educating nursing staff, given that only two of the nurses knew about the new regulations.

However, our findings also indicate that this might not be enough. We suggest that any efforts to develop new ways of acting must be grounded in an understanding of the importance of routines and habitus. The staff’s habitus has been incorporated over years while working under the same conditions, and has a directing influence on their strategies, considerations and beliefs [42]. Therefore, when implementing new regulations, work-place managers must consider staff’s habitus. To change this habitus, all staff need opportunities to learn and experience the new values. In situations of change, it is often easier for some people in a group to accept the new situation [42]. According to Scalzi et al., the presence of a critical mass of “change champions” with shared values and goals seems to have an influence on bringing about sustained change in nursing homes [6]. In this way, one can imagine that the habitus can gradually be changed, and the new regulations can be implemented in staff’s daily work.

Practice in nursing homes is very complex, because staff’s actions depend on many factors [43]. This complexity needs to be explored and described in further research into practical actions in nursing homes. In this paper, we have focused on how routines and the flow of daily work direct practical actions. There is a need for further exploration of this topic; for example, what happens if the routines and the workflow are disturbed?

### Strengths and limitations

A limitation of this study is that it was carried out in a Norwegian context. Another limitation is that data are based on observation by one researcher. Participant observation is subjective in its character and will be coloured by the researcher’s expectations. Therefore, the preliminary findings were discussed with the co-authors during the whole analytical process, before they were presented back to and discussed with the participants. Then, the findings were examined in the light of theory, to develop a possible understanding of the findings. To minimize the limitations, we have carefully described the context, data collection and analytical process.

## Conclusions

This study describes how daily routines and the carers’ habitus guide work in nursing homes. Staff actions seem to flow automatically throughout the day, and they just do them, without being able to describe why. The new regulations that were introduced are therefore a challenge to implement because routines and habitus are so strongly embedded in nursing practice. The study also reveals that routines are helpful and make the nursing home day predictable. Improving practice will require systematic and deeply rooted practical change in everyday actions and thinking.

## Competing interests

The authors declare that they have no competing interests.

## Authors’ contributions

AMS was responsible for the study conception and design, data collection, data analysis, and manuscript drafting. SH and KK were responsible for the study conception and design, and critical revisions of the manuscript. All authors read and approved the final manuscript.

## Pre-publication history

The pre-publication history for this paper can be accessed here:

http://www.biomedcentral.com/1472-6955/11/7/prepub
